# K-Y™ jelly inhibits increase in endotracheal tube cuff pressure during nitrous oxide exposure in vitro

**DOI:** 10.1186/s12871-018-0566-9

**Published:** 2018-07-28

**Authors:** Yukihide Koyama, Hiroyuki Oshika, Hiroko Nishioka, Naoko Kamoshida, Sousuke Tanaka, Gaku Inagawa, Tomio Andoh

**Affiliations:** 1 Department of Anesthesiology, Mizonokuchi Hospital, Teikyo University School of Medicine, 5-1-1 Futako, Takatsu-ku, Kawasaki, Kanagawa 213-8507 Japan; 2 Department of Medical Engineering, Mizonokuchi Hospital, Teikyo University School of Medicine, Kawasaki, Japan; 3 Department of Anesthesiology, Yokohama Municipal Citizen’s Hospital, Yokohama, Japan

**Keywords:** K-Y™ jelly, Lubrication, Tracheal tube cuff, Cuff pressure increase, Nitrous oxide diffusion

## Abstract

**Background:**

The increase in endotracheal tube cuff pressure due to nitrous oxide diffusion is a well-known risk during general anesthesia using nitrous oxide. We hypothesized that lubricating endotracheal tube cuffs with K-Y™ Jelly might inhibit the increase in cuff pressure that occurs during exposure to nitrous oxide.

**Methods:**

We used two types of endotracheal tube cuffs: one made from ultrathin polyurethane (PU) and another made from conventional polyvinyl chloride (PVC). Using a pediatric trachea model, which consisted of an acrylic cylinder with an internal diameter of 12 mm, we measured changes in the cuff pressure during nitrous oxide exposure in size 5.0-mm internal diameter endotracheal tubes with each type of cuff, with and without lubrication with K-Y™ Jelly.

**Results:**

During nitrous oxide exposure, the increase in cuff pressure was significantly lower in the lubricated cuffs than in the non-lubricated cuffs in both types of cuffs (PVC, *P* < 0.0001; PU, *P* < 0.0001). However, the cuff compliance in the trachea model was unaffected by lubrication in both types of cuffs.

**Conclusions:**

Lubrication of endotracheal tube cuffs with K-Y™ Jelly may effectively delay the increase in cuff pressure that occurs during general anesthesia using nitrous oxide.

## Background

The increase in endotracheal tube (ETT) cuff pressure due to nitrous oxide (N_2_O) diffusion is a well-known risk during general anesthesia using N_2_O [1–3]. N_2_O diffusion increases the cuff volume and pressure, causing an increased risk of tracheal barotrauma, the results of which range from sore throat to tracheal stenosis and tracheomalacia [4, 5]. Consequently, cuff pressure monitoring should be performed carefully under general anesthesia, particularly if N_2_O is used in clinical practice. Ischemic tracheal mucosal damage followed by edema after extubation is especially problematic in pediatric patients due to the smaller internal diameter (ID) of the trachea and the presumed lower perfusion pressure of the tracheal mucosa in children [3]. Therefore, the increase in ETT cuff pressure can be much more hazardous in pediatric patients than in adults.

It has been well documented in laboratory and clinical studies that lubrication of ETT cuffs reduces liquid and air leakage around the cuffs [6–9]. However, the effect of lubrication on changes in the ETT cuff pressure during N_2_O exposure has not been studied. Two types of ETT cuffs are currently available: those made from ultrathin polyurethane (PU) and those made from conventional polyvinyl chloride (PVC). Ultrathin PU cuffs are frequently used in infants because they allow for tracheal sealing at lower cuff pressures than conventional PVC cuffs [10]. K-Y™ Jelly (Johnson & Johnson, New Brunswick, NJ, USA), the major component of which is glycerin, is a commonly used lubricant in clinical settings. We hypothesized that a layer of glycerin on the ETT cuff surface would reduce N_2_O diffusion into the cuff and reduce the change in cuff pressure during exposure to N_2_O. In the present study, we tested this hypothesis using a rigid pediatric trachea model and the two types of ETT cuffs with or without lubrication with K-Y™ Jelly.

## Methods

**Fig. 1:**
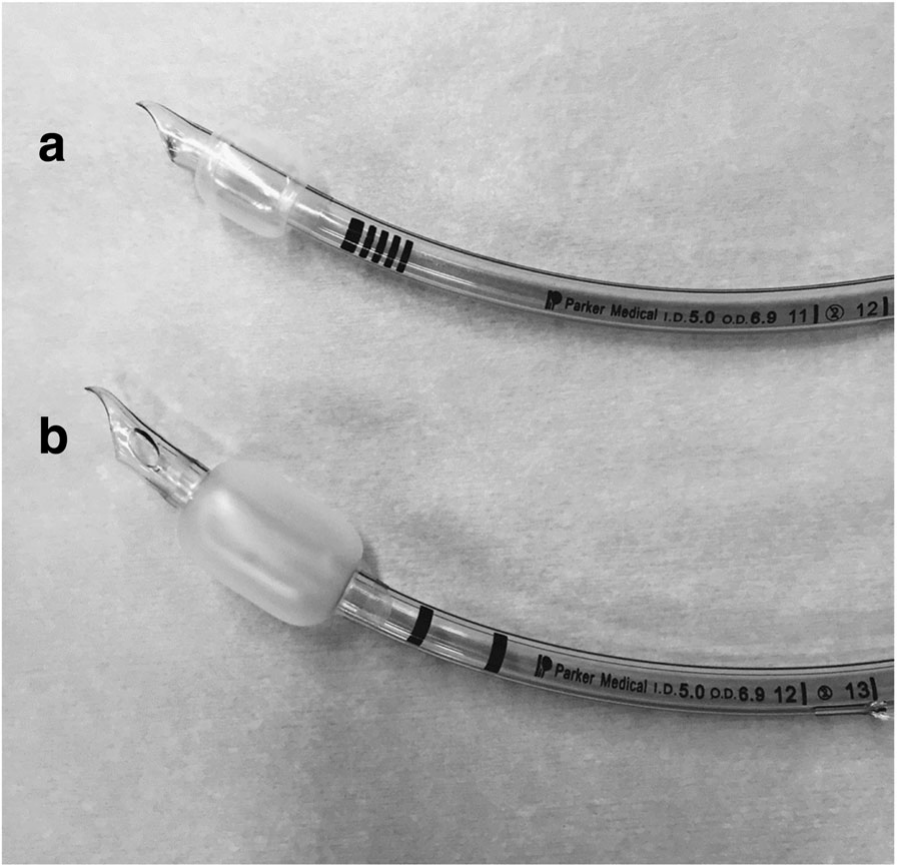
Visual comparison of the two types of cuffs inflated with air. **a** The cuff of the Parker ThinCuff™ Tracheal Tube is made from ultrathin polyurethane. **b** The cuff of the Parker Flex-Tip™ Tracheal Tube is made from conventional polyvinyl chloride

We used a Parker Flex-Tip™ tracheal tube for the PVC cuff and a Parker ThinCuff™ tracheal tube for the PU cuff (Parker Medical, Highlands Ranch, CO, USA), both of which had an ID of 5.0 mm (Fig. 1). For lubrication, 1.5 g of K-Y™ Jelly was used, as described in a prior study [9]. In the lubricated cuff group, after weighing out 1.5 g of K-Y™ Jelly on a pre-weighed plastic dish, ETT cuffs were fully lubricated by dipping and rotating ETT cuffs in the K-Y jelly. A pediatric trachea model, which consisted of an acrylic cylinder with an ID of 12 mm, was used to simulate the trachea of an approximately 8-year-old patient [9]. An acrylic cylinder has been used as a trachea model in prior studies [9, 11]. The trachea model was fixed horizontally and intubated with the ETTs with or without lubrication. Cuff pressures were measured with a pressure transducer (TruWave MP5100™, Edwards Lifescience, Irvine, CA, USA) connected to the pilot balloon and monitored on an anesthesia monitoring system (IntelliVew MP70™, Philips Medical System, Eindhoven, The Netherlands). The trachea model was connected to a test lung, and the ETT was connected to an anesthesia machine (Avance CS2 with ecoFLOW™, GE Healthcare UK Ltd., Buckinghamshire, England).

### Preliminary study

We reported in our earlier study that lubrication with K-Y™ Jelly improves air sealing of pediatric ETT cuffs [9]. For this reason, we were concerned that, in the absence of lubrication, ventilation may induce air leakage. Such air leakage could increase the surface area of N_2_O diffusion from the oral side of the cuffs, altering the results of cuff pressure changes. Therefore, we conducted a preliminary study to examine this possibility and to find a method to avoid this problem.

In the preliminary study, the cuff was inflated with air at a pressure of 15 mmHg (approximately 20 cmH_2_O). A gas sampling tube was then placed near the proximal side of the cuff to measure the N_2_O concentration and to detect air leakage under pressure-controlled ventilation with a peak airway pressure of 15 cmH_2_O, no positive end-expiratory pressure, and a frequency of 12/min using 3 L/min of 66% N_2_O in oxygen. The N_2_O concentration was measured with an anesthetic gas analyzer (M1019A Intelliview, Phillips, Andover, MA, USA). An apparent elevation of the N_2_O concentration was detected near the proximal side of both types of ETT cuffs without lubrication but was not detected near either type of ETT cuff with lubrication. However, we found no N_2_O leakage around either of the unlubricated ETT cuffs when they were continuously flushed at a constant airway pressure of 5 cmH_2_O using the same gas mixture.

### Stationary conditions

To avoid N_2_O diffusion on the oral side of the unlubricated cuff, we first employed the condition of continuous flushing rather than ventilation to measure cuff pressure changes during N_2_O exposure. We examined the cuff pressure behaviors in the PVC and PU cuffs in the absence and presence of lubrication under continuous flushing with 3 L/min of 66% N_2_O in oxygen at a constant airway pressure of 5 cmH_2_O. For the baseline measurement, the cuff was inflated with air to 15 mmHg. The cuff pressure was then recorded at 20 min, 40 min, and 60 min of N_2_O exposure.

After each experiment, we obtained the pressure-volume curve of each cuff and calculated the compliance of each cuff with and without lubrication in the rigid trachea model. For these purposes, the cuff was completely deflated in the rigid trachea model, and air was then slowly injected into the cuff using a syringe with a 3-way stop-cock. Once the cuff pressure reached 15 mmHg, air was further injected in increments of 0.2 ml, and the cuff pressure was recorded after each additional 0.2 ml of air up to the total amount of injected air of 1.0 ml. Compliance represents the change in volume divided by the change in pressure; therefore, compliance was calculated as follows: compliance (μL /mmHg) = 1000 μL (1.0 mL) / (the cuff pressure (mmHg) after 1.0 mL of air injection – 15 mmHg).

### Ventilation conditions

To compare the cuff pressure behavior in a ventilatory condition similar to realistic clinical situations, we measured the cuff pressure changes in each type of cuff with and without lubrication during pressure-controlled ventilation with a peak airway pressure of 15 cmH_2_O, no positive end-expiratory pressure, and a frequency of 12/min using 3 L/min of 66% N_2_O in oxygen (the same gas protocol used in stationary conditions). We then compared the results in each type of cuff in the absence and presence of lubrication under the condition of ventilation. Since Dullenkopf et al. performed 4 measurements for each experiment [3, 12], we also conducted each experiment 4 times, using a new ETT for each experiment. That is, a total of 32 experiments were performed, and the total number of new ETTs used in the present study, including those used in Experiments 1 and 2, was 32. All tests in the present study were performed at a room temperature of 24 °C.

### Statistical analysis

A two-way repeated measures analysis of variance (ANOVA) (time × lubricant) followed by an unpaired *t*-test with Bonferroni correction was used to compare the cuff pressure behaviors with and without lubrication in the PVC and PU cuffs under continuous flushing and ventilation. In addition, we analyzed the time-dependent changes in cuff pressure with each type of cuff for each condition using a one-way repeated measures ANOVA followed by a Dunnett’s test. Bonferroni correction was applied in the post hoc tests comparing cuff pressures between groups at each time point. *P*-values < 0.0167 (0.05/3) were considered statistically significant in the post hoc test at each time point. Cuff compliance with and without lubrication was compared using an unpaired *t*-test. Comparisons of the pressure-volume curves with and without lubrication in each type of cuff were also carried out by using a two-way repeated measures ANOVA (added air volume × lubricant).

## Results

**Fig. 2:**
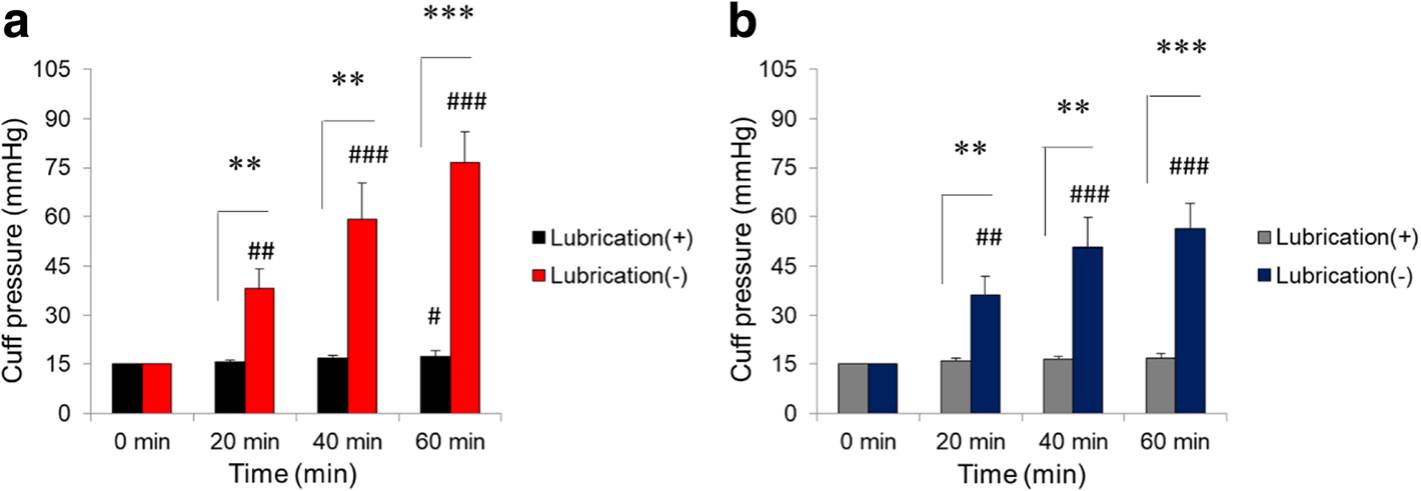
Comparison of cuff pressures with and without lubrication under continuous flushing with nitrous oxide. Black and red bars in Graph **a** represent polyvinyl chloride (PVC) cuffs with lubrication and PVC cuffs without lubrication under continuous flushing with N_2_O. Gray and blue bars in Graph **b** represent polyurethane (PU) cuffs with lubrication and PU cuffs without lubrication under continuous flushing with N_2_O. Cuff pressure was recorded at 0 min (baseline), 20 min, 40 min, and 60 min of nitrous oxide exposure. # *P* < 0.05, ## *P* < 0.01, and ### *P* < 0.0001 relative to the baseline (0 min) pressure at each time point. ** *P* < 0.001 and *** *P* < 0.0001 in the post hoc comparisons between 2 groups at each time point. *P*-values < 0.0167 (0.05/3) were considered statistically significant in the post hoc comparison of 2 groups at each time point. Data are expressed as mean (SD)

In stationary conditions, cuff pressure increased with time in both the PVC (*P* < 0.0001; F = 49.1) and PU cuffs (*P* < 0.0001; F = 34.8), and this increase was higher without lubrication in both the PVC (P < 0.0001; F = 246.3) and PU cuffs (P < 0.0001; F = 208.1). The interaction between time and lubrication was significant in both types of cuffs. The P and F values for the interaction were P < 0.0001; F = 41.6 for PVC cuffs and P < 0.0001; F = 29.4 for PU cuffs. Without lubrication, the cuff pressure significantly increased in a time-dependent manner in both types of cuffs (PVC, *P* < 0.0001; PU, *P* < 0.0001). In the lubricated PVC cuffs, a slight but significant increase in cuff pressure occurred over time (*P* = 0.022). In the lubricated PU cuffs, however, the increase in cuff pressure over time was not significant (*P* = 0.114). Lubrication with K-Y™ Jelly strongly inhibited the increase in cuff pressure in PVC cuffs and totally abolished the cuff pressure increase in PU cuffs. Without lubrication, the cuff pressures at the 20 min, 40 min, and 60 min time points were significantly greater than the cuff pressure at baseline (0 min) in both the PCV and PU cuffs (*P* < 0.01, *P* < 0.0001, and *P* < 0.0001, respectively)*.* Conversely, the increase in cuff pressure in the presence of lubrication was insignificant compared to the baseline except for the 60-min time point for the PCV cuffs. The cuff pressures without lubrication were significantly greater than those with lubrication at the 20 min, 40 min, and 60 min time points in both the PVC and PU cuffs (*P* < 0.001, *P* < 0.001, and *P* < 0.0001, respectively). These results are shown in Fig. 2.

**Fig. 3:**
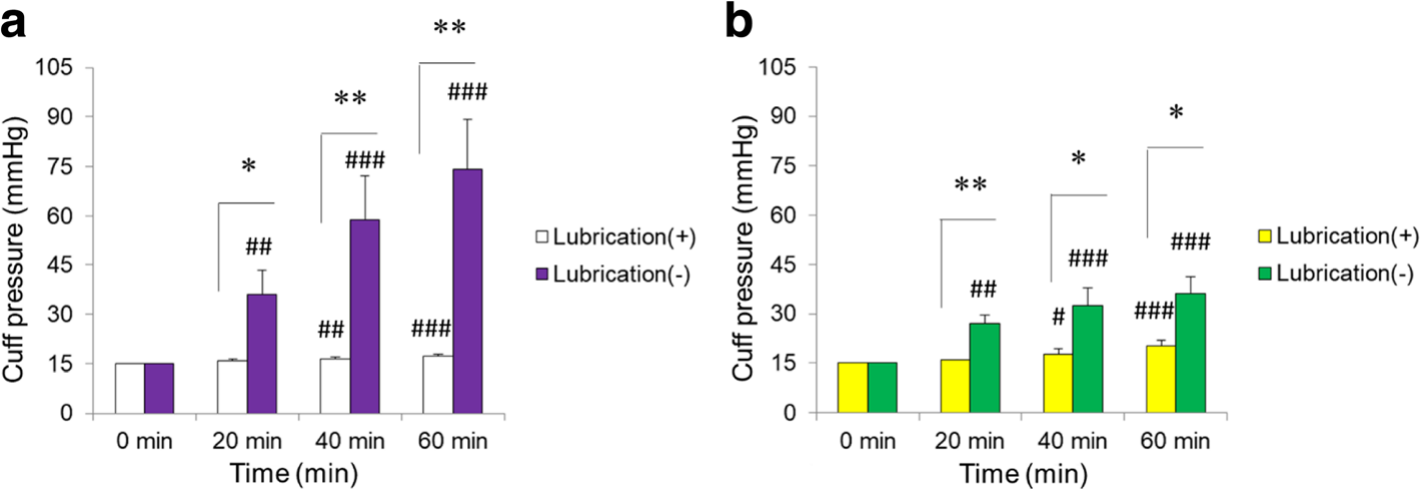
Comparison of cuff pressures with and without lubrication during nitrous oxide ventilation. White and purple bars in Graph **a** represent polyvinyl chloride (PVC) cuffs with lubrication and PVC cuffs without lubrication during ventilation with N_2_O. Yellow and green bars in Graph **b** represent polyurethane (PU) cuffs with lubrication and PU cuffs without lubrication during ventilation with N_2_O. Cuff pressure was recorded at 0 min (baseline), 20 min, 40 min, and 60 min of nitrous oxide exposure. # *P* < 0.05, ## *P* < 0.01, and ### *P* < 0.0001 relative to the baseline (0 min) pressure at each time point. * *P* < 0.01 and ** *P* < 0.001 in the post hoc comparisons between 2 groups at each time point. *P* values < 0.0167 (0.05/3) were considered statistically significant in the post hoc comparison of 2 groups at each time point. Data are expressed as mean (SD)

In ventilation conditions, application of K-Y™ Jelly strongly inhibited cuff pressure increase in both types of cuffs during ventilation with N_2_O. The interaction between time and lubrication was significant in both types of cuffs. The P and F values for the interaction were *P* < 0.0001; F = 21.1 for PVC cuffs and *P* < 0.0001; F = 12.0 for PU cuffs. Without lubrication, the cuff pressure significantly increased in a time-dependent manner in both types of cuffs (PVC, *P* < 0.0001; PU, *P* < 0.0001). Without lubrication, the cuff pressures at the 20 min, 40 min, and 60 min time points were significantly greater than the cuff pressure at baseline (0 min) in both types of cuffs (*P* < 0.01, *P* < 0.001 and *P* < 0.001, respectively). In both the lubricated PVC and PU cuffs, there was a significant increase in cuff pressure over time during ventilation (PVC, *P* < 0.0001; PU, *P* < 0.0001). With lubrication, the cuff pressures at the 40 min and 60 min time points were significantly greater than the cuff pressure at baseline (0 min) in both the PVC and PU cuffs (PVC, *P* < 0.01 and *P* < 0.0001; PU, *P* < 0.05 and *P* < 0.0001). Importantly, however, cuff pressures without lubrication were also significantly greater than those with lubrication at the 20 min, 40 min and 60 min time points in both the PVC and PU cuffs during ventilation (PVC, *P* < 0.01, *P* < 0.001, and *P* < 0.001; PU, *P* < 0.001, *P* < 0.01 and *P* < 0.01). These results are shown in Fig. 3.

**Fig. 4:**
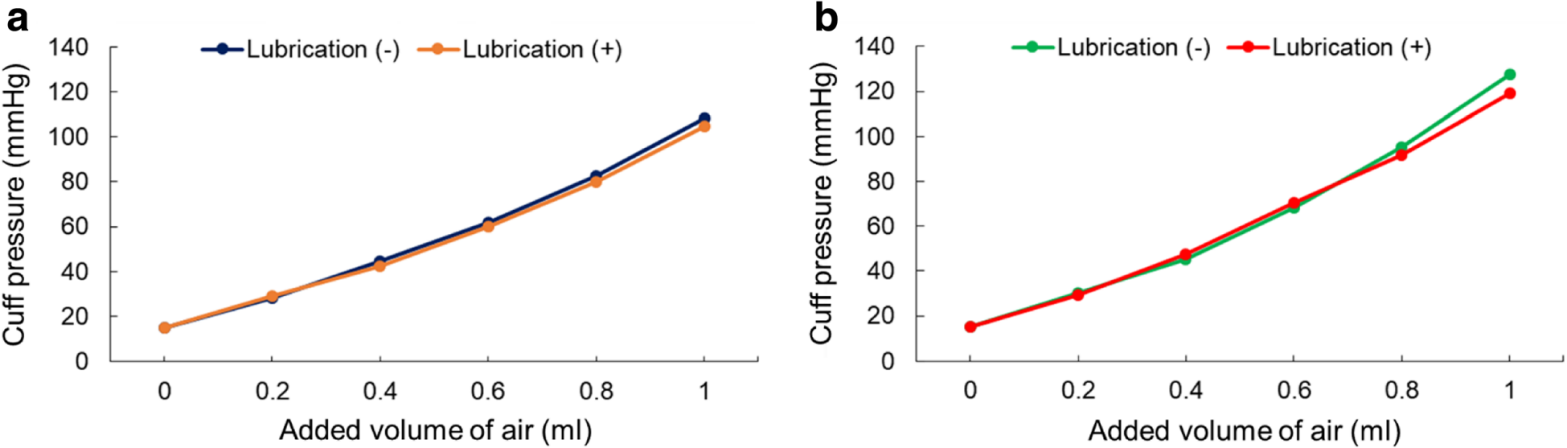
Pressure-volume curve with and without lubrication in PVC and PU cuffs (rigid trachea model). Pressure-volume curves of tested tracheal tube cuffs with and without lubrication restricted in a rigid trachea model. Graphs **a** and **b** show the results of the PVC and PU cuffs, respectively. Data are expressed as mean

Lubrication had no statistically significant effect on cuff compliance with either type of cuff in the rigid trachea model (Table 1). There was no significant interaction between added air volume and lubrication in either the PVC (*P* = 0.652; F = 0.62) or PU cuffs (*P* = 0.404; F = 1.04) (Fig. 4). Lubrication induced no significant changes in the pressure-volume relationships of either the PVC (*P* = 0.53; F = 4.1) or PU cuffs (*P* = 0.38; F = 0.789) (Fig. 4).

**Table 1:** Cuff compliance in conventional polyvinyl chloride (PVC) cuffs and ultrathin polyurethane (PU) cuffs with and without lubrication in the rigid trachea model

	Lubrication (+)	Lubrication (−)	*P* Value
Cuff compliance (μL /mmHg) PVC	11.0 ± 0.5	10.7 ± 0.3	0.36
Cuff compliance (μL /mmHg) PU	9.6 ± 0.2	9.4 ± 0.2	0.13

Data are expressed as mean (SD). Cuff compliance (μL/mmHg) = ΔVolume (μL) / ΔPressure (mmHg)

## Discussion

Cuffed ETTs are being increasingly used in pediatric anesthesia, as studies have shown that they are as safe as uncuffed ETTs for children undergoing general anesthesia [13–17]. Although the use of N_2_O is becoming less frequent, it is still commonly used as part of general anesthesia world-wide. In the United States, N_2_O was included in approximately 35% of all general anesthesia cases reported to the Anesthesia Quality Institute [18]. Furthermore, the long-term safety of N_2_O administration during general anesthesia has been well-documented in large clinical trials [18, 19]. Thus, N_2_O is still an important part of general anesthesia. Therefore, management of the ETT cuff pressure during general anesthesia using N_2_O is clinically important and of great concern in pediatric anesthesia.

In the present study, we found that pretreatment of both PVC and PU cuffs with K-Y™ Jelly strongly and significantly inhibited the increase in cuff pressure that occurred during N_2_O exposure in a pediatric trachea model during continuous flushing and pressure-controlled ventilation with 66% N_2_O in oxygen. To the best of our knowledge, this is the first study to investigate how lubrication of ETT cuff affects cuff pressure behavior during N_2_O exposure. To avoid the effect of N_2_O diffusion on the oral side of the cuff due to air leakage in the absence of a lubricant, we first employed continuous flushing of the ETT with N_2_O at 5 cmH_2_O of constant airway pressure rather than ventilation in stationary conditions. Our results showed that during N_2_O exposure, the cuff pressure in the absence of lubrication significantly increased over time even in the absence of ventilation. This finding is consistent with the results of a previous study in which ventilation was performed during measurements [20]. However, we could not exclude the possibility that ventilation through the ETT might influence the cuff pressure behavior during N_2_O exposure by removing lubricants from the cuff over time and by changing the N_2_O concentration on the carina side of the cuff. Consequently, we also compared the cuff pressure changes during ventilation to study the potential effects of ventilation on the lubrication-induced inhibition of cuff pressure increases during N_2_O exposure in ventilation conditions.

Lubrication also strongly and significantly inhibited the cuff pressure increase in each type of cuff when ventilation with N_2_O was done. In ventilation conditions, N_2_O diffusion into the oral side of the cuff may have occurred only in non-lubricated cuffs. This may have been due to the apparent elevation of the N_2_O concentration detected near the oral side of both types of ETT cuffs without lubrication during ventilation. However, the leakage must have been small, because the anesthesia machine did not detect air leakage in the anesthesia circuit. We confirmed that the inhibitory effect of lubrication on the cuff pressure increase caused by N_2_O diffusion was maintained in each type of cuff in the presence of ventilation in vitro.

Lubrication altered neither the compliance nor the pressure-volume relationships of either type of ETT cuff placed in the rigid trachea model. Therefore, we raise the possibility that the layer of glycerin-based K-Y™ Jelly inhibited the cuff pressure increase by reducing the diffusion of N_2_O into the cuff.

ETT cuff pressure monitoring during general anesthesia using N_2_O is recommended to ensure that the cuff pressure remains within safe limits to avoid airway morbidity in children [21, 22] and adults [12, 20]. It is standard clinical practice to monitor and adjust the cuff pressure carefully during general anesthesia using N_2_O. Slowing the cuff pressure increase by lubricating the ETT may have limited clinical significance because this does not eliminate the necessity of cuff pressure monitoring. However, lubricating the cuffs may decrease the frequency of needing to adjust the cuff pressure or eliminate the need to adjust cuff pressure during short surgeries under general anesthesia using N_2_O. Thus, ETT lubrication with K-Y ™ Jelly may reduce the incidence of complications associated with cuff pressure manipulation, such as the cough reflex, bucking, and hemodynamic responses like tachycardia and hypertension.

The present study does have some limitations. First, because the rigid trachea model is much more rigid than an actual human trachea, the rate of cuff pressure increase during N_2_O exposure might be slower in clinical settings than in the laboratory. Therefore, we cannot exclude the possibility that the inhibitory effect of lubrication with K-Y™ Jelly on the N_2_O-induced increase in cuff pressure is potentially overestimated in the present study.

Secondly, to extrapolate our findings to the clinical setting, we need to consider possible differences between in vitro and clinical conditions. For example, the amount of K-Y™ Jelly retained on the cuff may be reduced during tracheal intubation, suggesting that the effect of lubrication may be decreased in the clinical setting. Additionally, the water-soluble lubricant, K-Y™ Jelly may be washed out by mucus derived from the tracheal membrane. Therefore, the inhibitory effects of the lubricant on the cuff pressure increase may not last for a long time in clinical settings. However, it has been shown that the inhibition of fluid leakage across tracheal tube cuffs by K-Y ™ Jelly lasts for about 24 h in adult patients [6]. This finding suggests that a part of K-Y™ Jelly may remain on the cuff for much longer than the duration required for common surgical procedures in clinical situations. Furthermore, N_2_O diffusion occurs through the tracheal wall membrane in clinical settings [12]. Consequently, lubrication may be less effective in inhibiting N_2_O diffusion into the cuff from the tracheal membrane because the layer of the lubricant on the cuff surface in contact with the tracheal membrane may be thinner than the layer of lubricant on the carina side of the cuff.

Thirdly, temperature has a large effect on the transport of penetrants in the polymeric media [23]. In some polymers, gas permeability increases with increase in temperature [23]. Furthermore, Brandt, et al. [24] demonstrated that ETT cuff pressure under N_2_O anesthesia can change with body temperature. As a result, we cannot exclude the possibility that gas permeability in the glycerin-based K-Y™ Jelly might increase with temperature, a behavior similar to some polymers [23]. In the present study, we conducted all tests at 24 °C, which is much lower than body temperature. Therefore, it is unclear how lubrication influences the cuff pressure increase during N_2_O exposure at body temperature. However, we believe that our results are sufficiently encouraging to conduct a clinical study in human patients.

## Conclusions

The results of the present study demonstrate that lubrication of ETT cuffs with K-Y™ Jelly strongly inhibits the increase in cuff pressure during N_2_O exposure in a pediatric trachea model. These findings suggest that lubrication of the cuff may effectively delay the increase in cuff pressure that occurs during general anesthesia using N_2_O. Further studies are warranted to test whether these findings hold true in the clinical setting.

## Abbreviations

ETT: Endotracheal tube
ID: Internal diameter
N_2_O: Nitrous oxide
PU: Polyurethane
PVC: Polyvinyl chloride
